# Use of the GTT@home Oral Glucose Tolerance Test Kit in Gestational Diabetes Mellitus: Performance Evaluation Study

**DOI:** 10.2196/69695

**Published:** 2026-02-11

**Authors:** Gareth J Dunseath, Stephen D Luzio, Wai Yee Cheung, Sharon N Parsons, Nicola John, Mahmoud Chokor, Michael Atkinson, Rajesh Peter

**Affiliations:** 1 Diabetes Research Group, Swansea University, Ground Floor, Grove Building, Singleton Campus, Swansea, SA28PP, United Kingdom, 44 1792513439; 2 Neath Port Talbot Hospital, Port Talbot, United Kingdom

**Keywords:** device, diagnosis, gestational diabetes, OGTT, glucose, oral glucose tolerance test

## Abstract

**Background:**

The 75-g oral glucose tolerance test (OGTT) remains the optimal diagnostic test for use in pregnancy but needs to be performed in the clinical setting. The GTT@home OGTT device offers the potential to enable patients to perform the test at home using capillary blood samples.

**Objective:**

This study aimed to determine the accuracy of the GTT@home device compared to the routine National Health Service laboratory reference method using blood samples during an OGTT from pregnant women at high risk of developing gestational diabetes mellitus (GDM).

**Methods:**

A total of 65 women (aged >18 y), at high risk for GDM (per the National Institute for Health and Care Excellence guidelines) were recruited for this performance evaluation. Following an overnight fast, participants went for a 75-g OGTT. Fasting and 2-hour capillary glucose levels were measured using the GTT@home device with corresponding venous samples measured in the laboratory.

**Results:**

The complete data for analysis was available for 61/65 devices. The overall bias for the GTT@home device was +0.16 mmol/L. Correlation analysis of the clinical performance of the two methods using a surveillance error grid showed 79.8% of results in the lowest, 16.9% in the “slight, lower” and 2.4% in the “slight, higher” risk categories. Only 0.8% were “moderate, lower” risk, and none were in any higher risk categories. There was agreement in the classification in 54/61 cases. The GTT@home device under-classified 2 cases and over-classified 5 cases.

**Conclusions:**

The GTT@home device worked well in a controlled, antenatal clinical setting. Differences in classification observed were generally due to small differences in glucose values close to the diagnostic cut-offs. The GTT@home device shows promise for home testing of glucose tolerance in pregnant women.

## Introduction

Gestational diabetes mellitus (GDM) is a common metabolic disorder occurring in up to 10% of pregnancies in the Western world [1]. Most women with GDM are asymptomatic, and therefore, it is important to screen, diagnose, and manage the condition, as it is associated with an increased risk of maternal and perinatal complications such as pre-eclampsia, macrosomia, shoulder dystocia, and neonatal hypoglycemia. In the United Kingdom, in line with the National Institute for Health and Care Excellence (NICE) guidelines, women with a high risk of GDM are offered a 75-g oral glucose tolerance test (OGTT) at 24‐28 weeks gestation [2], with plasma glucose levels ≥ 5.6 mmol/l or 2-hour plasma glucose levels ≥ 7.8 mmol/l being diagnostic of GDM.

Unlike diabetes mellitus in the general population, where the 2-hour 75-g OGTT has largely been superseded by the glycosylated hemoglobin level for diagnosis, the OGTT carried out in a clinical setting is still the optimal test for use in pregnancy. The glycosylated hemoglobin level is not sufficiently sensitive to substitute for the OGTT as a screening test, due to the variations in red blood cell turnover seen in pregnancy. However, during the COVID-19 pandemic, changes to diagnosis of GDM were forced on maternity services, with diagnosis being determined by the measurement of either glycosylated hemoglobin or a single fasting or random plasma glucose sample. A retrospective study of prospectively collected data [3] has shown that using the Royal College of Gynecologists COVID-19 Gestational Diabetes screening guideline failed to detect 47 of 82 (57%) women subsequently identified with GDM and therefore could not be recommended for general use. A scoping review of the guidelines and diagnostic studies evaluating the recommended testing strategies [4] concluded that the OGTT remains the most effective test to identify abnormal glucose tolerance in pregnancy.

In practice, pre-analytical processing of blood samples can affect plasma glucose concentrations due to continuing glycolysis by red cells prior to centrifugation [5-11]. The American Diabetes Association and American Association of Clinical Chemistry recommend that samples for plasma glucose measurement should be collected into sodium fluoride tubes and placed in an ice-water slurry prior to centrifugation within 30min [12,13]. If a delay in centrifugation is anticipated, citrate tubes should be used as citrate more rapidly inhibits glycolysis [12,13]. In routine clinical practice, this is rarely carried out, which could affect the diagnosis of GDM.

The GTT@home OGTT device is an electronic device that has the potential to enable patients to perform an OGTT from home using capillary blood samples. In non-pregnant women, the GTT@home device has been previously shown to be easy to use, reliable and demonstrated excellent agreement with the results obtained from a reference laboratory analyzer (YSI 2300 stat Plus) [14]. It now needs to be established how results from this device compare with results obtained conventionally from an OGTT in women at risk of GDM. In this study, glucose concentrations during an OGTT were tested with fresh blood samples from women at risk of GDM in the United Kindgom and compared to routine laboratory glucose concentrations.

## Methods

### Ethical Considerations

Women gave written informed consent to take part in the study. Ethical approval was obtained from Health Research Authority and Health and Care Research Wales, Wales REC 6 (22/WA/0153). Patients or the public were not involved in the design, or conducting, reporting, or dissemination plans of our research. All participant data were anonymized prior to analysis. Participants received no compensation for taking part in this study.

### Participants

This performance evaluation study was carried out with women presenting to the antenatal clinic at Neath Port Talbot Hospital for a routine 75-g OGTT at approximately 24‐28 weeks of gestation. Study participants (n=65) were female, aged >18 years, were at high risk for developing GDM according to NICE guidelines (ie, previous macrosomic baby weighing >4.5 kg or >90th centile, previous GDM, family history of diabetes [first-degree relative with diabetes], or an ethnicity with a high prevalence of diabetes). Participants were excluded if they were unable or unwilling to give informed consent.

### GTT@home Device

**Figure 1.**
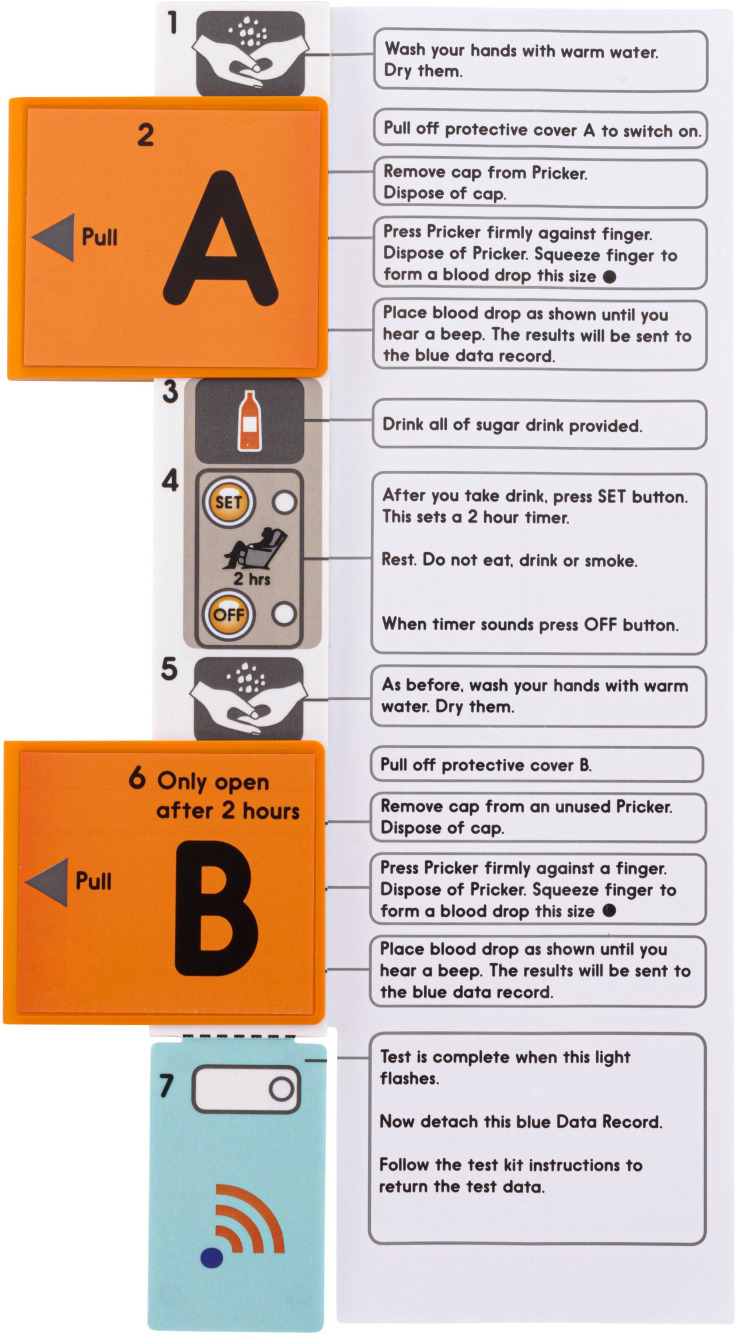
Example of the GTT@home device including user instructions. GTT: glucose tolerance test.

The Home OGTT devices were provided by Digostics Ltd (Figure 1). The device consists of 2 glucose dehydrogenase test strips (0 and 2-h) with user activated buttons. The test procedure was driven by an integral clock and timer, with audible and visual prompts. Each single-use, disposable device was stored in sealed packaging and opened immediately prior to use. The device was activated by removing a protective cover over the 0-hour test strip (A) and the capillary blood sample being placed on the test strip. Following consumption of the glucose drink, the “set” button is pressed to begin the timer, and after 2 hours, an audible alarm alerts the user to press “stop” and repeat the sampling process with the 2-hour test strip (B). A further audible alarm confirms the test was complete. A detachable data recorder is scanned, and the result automatically transferred to a secure web-based database. No results are visible to the user.

### Study Procedure

Participants attended the antenatal clinic at Neath Port Talbot Hospital on one occasion having fasted for 8 hours. At time 0, a finger prick blood sample was collected using a finger pricking device. The blood drop was applied to the 0 minutes (A) glucose sensor on the GTT@home device. A venous blood sample (2 ml) was also taken for routine hospital laboratory analysis as per usual practice (ie, the Roche Cobas enzymatic method). Following the 0-min sample, a drink containing 75 g glucose was given to the subject. At 2 hours following the glucose drink, a second finger prick and venous blood sample were collected. The finger prick capillary blood was applied to the 2-hour (B) sensor on the GTT@home device, and the venous blood was sent to the routine hospital laboratory for blood glucose measurement. Following the 2-hour sample, the participants were allowed to leave the clinic.

The readings from the two sets of tests were generated independently in different locations. Assessors were not able to be influenced by prior knowledge of readings from the other test. Clinical staff collecting the clinical information and performing the GTT@home test were blinded to these results.

The GTT@home device readings were generated automatically and were non-editable. Results were stored electronically on a detachable data chip, scanned, and uploaded to a password-protected database.

### Data Analysis

Based on a GDM prevalence of 35%, to achieve the minimally acceptable kappa score of 0.61, a sample size of 65 patients was required to achieve 80% power and a statistical significance of 0.05.

Bias was assessed using Bland-Altman plots of the GTT@home device versus the routine hospital laboratory method [15]. Correlation of the clinical performance of the two methods was assessed using a surveillance error grid [16]. Agreement of the diagnoses generated using the GTT@home device and the routine laboratory analyzer with the reference was assessed using receiver operating characteristic curves (sensitivity and specificity) and positive and negative predictive values [17]. Agreement between the readings was assessed using kappa analysis for the whole OGTT, fasting, and 2-hour glucose values. Patients were categorized as having normal glucose tolerance or being intolerant (0 or 1).

The study classified the status of glucose tolerance according to the NICE criteria [1] with glucose intolerance defined as a fasting plasma glucose level ≥5.6 mmol/l and/or a 2-hour plasma glucose level ≥7.8mmol/l.

## Results

The mean (SD) age of participants was 29.6 (4.8; range 19-41) years, and the mean (SD) BMI was 32.8 (7.6; range 16.5-54.7) kg/m^2^ (Table 1).

Results from 4 participants were not included in the final analysis: two devices reported only fasting glucose values, one device reported no results, and one patient experienced nausea, so the venous sample was not collected.

**Figure 2.**
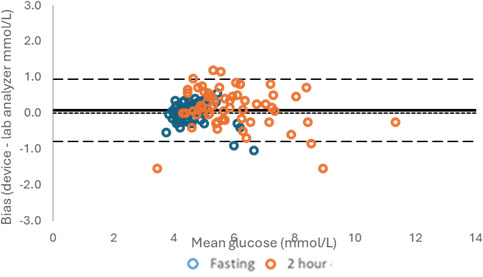
Bias of the GTT@home device. The blue circles indicate fasting levels, while the orange circles indicate the levels after 2 hours.

The bias of the GTT@home device compared to the routine laboratory method is shown in Figure 2. For fasting plasma glucose, the bias (lower/upper limit of agreement) was 0.01 (−1.13/1.15) mmol/L; at 2 hours, it was +0.31 (-1.84/2.46) mmol/L; and for all results, the bias was +0.16 (−1.57/1.89) mmol/L.

**Figure 3.**
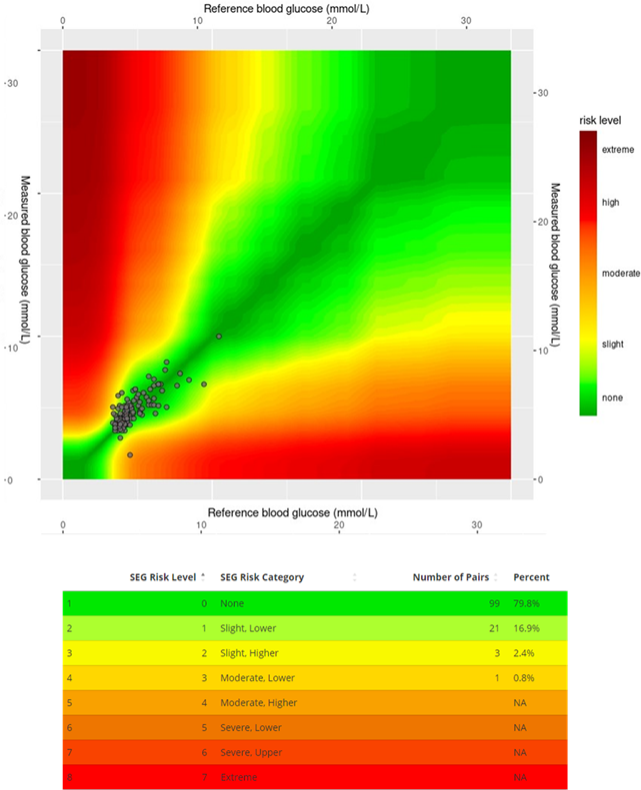
Surveillance error grid (SEG).

Correlation of the clinical performance of the two methods is shown in Figure 3 using a surveillance error grid. A total of 79.8% of results were in the lowest risk category (“none”), 16.9% in the “slight, lower” risk category, and 2.4% in the “slight, higher” risk category. Only 0.8% were in the “moderate lower” risk category, and there were no results in any of the higher risk categories.

**Table 1.** Demographic characteristics.

Demographic characteristic	Mean (SD)	Range
Age (y)	29.6 (4.8)	19.0-41.0
Height (m)	1.64 (0.1)	1.53-1.83
Weight (kg)	89.3 (23.1)	40.0-148.0
BMI (kg/m^2^)	32.8 (7.6)	16.6-54.7

Cross-tabulation of overall classification (combined fasting and 2 h) is shown in Table 2. There was overall agreement in classification in 54 out of 61 cases. The GTT@home device under classified 2 cases and over classified 5 cases. Of these small number of different classifications, the majority were close to the diagnostic thresholds, with one-third fasting plasma glucose results and two-third 2-hour results within 0.2 mmol/L of the respective cut-offs.

**Figure 4.**
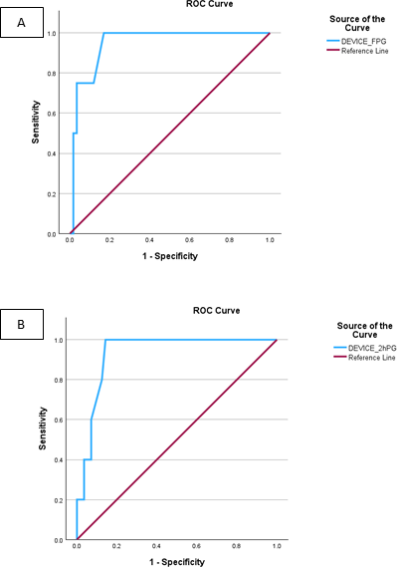
Receiver operating characteristic (ROC) curves with sensitivity/specificity. (A) Fasting glucose (cut-off 5.6 mmol/L); (B) 2-hour glucose (cut-off 7.8 mmol/L). FPG: fasting plasma glucose.

Receiver operating characteristic curves are shown in Figure 4, and positive and negative predictive values in Table 3. The AUC, sensitivity and specificity using receiver operating characteristic analysis for fasting glucose was 0.947, 0.75 and 0.966, respectively, and those for the 2-hour glucose were 0.932, 0.4 and 0.929, respectively. The kappa statistic for the GTT@home device compared to routine laboratory measurement was 0.457 (0.641 for fasting values and 0.301 for 2-h values). The diagnosis of glucose intolerance using both OGTT concentrations showed a positive predictive value of 0.44 and negative predictive value of 0.96.

**Table 2.** Cross tabulation.

		GTT@home device		Total
		NGT^a^	GDM^b^	
Overall
Laboratory	NGT	50	5	55
	GDM	2	4	6
Total	—^c^	52	9	61
kappa	0.457			
Fasting
Laboratory	NGT	57	2	59
	GDM	1	3	4
Total	—	58	5	63
kappa	0.641			
2-hour
Laboratory	NGT	52	4	56
	GDM	3	2	5
Total	—	55	6	61
kappa	0.301			

a NGT: normal glucose tolerance.

b GDM: gestational diabetes mellitus.

c Not applicable.

**Table 3.** Positive and negative predictive values.

	Positive predictive value	Negative predictive value
Overall	0.44	0.96
Fasting glucose	0.6	0.98
2-hour glucose	0.33	0.95

## Discussion

### Principal Findings and Comparison With Previous Works

In pregnant women in a clinical setting, the GTT@home OGTT device was compared with routine laboratory analysis of blood glucose samples. The GTT@home device worked well with relatively few failures. It showed a low bias (+0.16 mmol/L) across the range of all glucose concentrations, and for both fasting (+0.01 mmol/L) and 2-hour (+0.31 mmol/L) samples. Comparison of the classification of glucose intolerance between the GTT@home device and measurement using conventional laboratory glucose analysis showed small differences in the numbers of participants in the glucose intolerance categories.

These small differences may well reflect the tolerance around the different glucose analytical methods used. A previous study in which samples were analyzed at five central laboratories using four different automated glucose hexokinase methods demonstrated that despite there being low bias in glucose measurements across laboratories, the resulting GDM prevalence ranged considerably from 30.0% to 41.1% across laboratories [18]. Furthermore, even within the hospital clinical setting, the turn-around time of glucose samples can impact the clinical accuracy of laboratory measurements. Jangam et al [19] observed that delays of 15 min or more reduced clinical accuracy below 95%, and the accuracy was less than 65% for delays of 60 min. These processing delays in glucose measurements reduced the clinical relevance of results in patients with type 1 diabetes and were likely to similarly degrade the clinical value of measurements in other patient populations.

In community settings, pre-analytical issues and possible degradation of samples during transfer to the laboratory can severely affect the rates of diagnosis of GDM. Jamieson et al [20] showed an underdiagnosis rate of 62% due to the impact of long delays in centrifugation for OGTT samples in regional, rural, and remote sites in Western Australia. Potter et al [11] also showed that the variability in pre-analytic processing of blood for glucose measurement during pregnancy OGTTs could affect the GDM diagnostic rates, observing an increase in the rate of GDM from 11.6% to 20.6% by changing the process to centrifuging blood collected into sodium fluoride tubes, within 10 minutes of venipuncture.

In the context of our study, the relatively poor kappa statistics and positive predictive value score are likely to be due to the low number of participants with GDM included in the study cohort, small differences in glucose values close to the diagnostic cut-offs, and possibly due to the narrow range of glucose concentrations tested. In addition, pre-analytical issues and possible degradation of samples during transfer to the laboratory may also be responsible, as reflected by the device classifying slightly more individuals as having GDM than the laboratory method.

### Conclusions

The GTT@home device worked well in a controlled, antenatal clinical setting. Differences in classification observed were likely due to pre-analytical issues associated with the laboratory tested samples. The GTT@home device therefore shows promise for home testing of glucose tolerance in pregnant women, in addition to wider community use. Further insight into the real-life usage of the device will be achieved with future studies by comparing an at-home diagnosis using the GTT@home device, performed by the OGTT recipient, within a few days of a routine in-hospital laboratory-measured OGTT.

## Abbreviations

GDM: gestational diabetes mellitus
NICE: National Institute for Health and Care Excellence
OGTT: oral glucose tolerance test
